# RETRACTION: **Comparison of the Effect of Dydrogesterone and Natural Micronized Progesterone for Luteal‐Phase Support in Assisted Reproductive Technology Cycles: A Single‐Blind Randomized Clinical Trial Study**

**DOI:** 10.1002/hsr2.71139

**Published:** 2025-08-05

## Abstract

**RETRACTION:** M. Atarieh, M. Javadian, Z. Basirat, M. Kashifard, S. Yazdani, H. Adib‐Rad, M. Abdollahzade‐Delavar, and H. Gholinia, *Health Science Reports* 7, no. 8 (2024): e2296. https://doi.org/10.1002/hsr2.2296.

The above article, published online on 08 August 2024 in Wiley Online Library (wileyonlinelibrary.com), and has been retracted by agreement between the journal Editor‐in‐Chief, Dr. Charles Young; and Wiley Periodicals LLC. A third party reported concerns about the data integrity, methodological transparency, and reporting inconsistencies in this article, including inconsistencies in several datasets and several instances of missing model parameters which prevent verification of the results. An independent review conducted by the journal verified the majority of these concerns and found that some results were not supported by the available data.

The authors responded to an inquiry by the publisher by addressing the reported concerns and providing an amended manuscript which included the missing study design and methodological details. Crucially, the corrected manuscript included changes which fundamentally altered the results and conclusions of the article. All parties have agreed that the article must be retracted because the results and conclusions presented in the article are no longer considered reliable. The authors The authors were informed of the retraction.

**RETRACTION:** M. Atarieh, M. Javadian, Z. Basirat, M. Kashifard, S. Yazdani, H. Adib‐Rad, M. Abdollahzade‐Delavar, and H. Gholinia, *Health Science Reports* 7, no. 8 (2024): e2296. https://doi.org/10.1002/hsr2.2296.

The above article, published online on 08 August 2024 in Wiley Online Library (wileyonlinelibrary.com), and has been retracted by agreement between the journal Editor‐in‐Chief, Dr. Charles Young; and Wiley Periodicals LLC. A third party reported concerns about the data integrity, methodological transparency, and reporting inconsistencies in this article, including inconsistencies in several datasets and several instances of missing model parameters which prevent verification of the results. An independent review conducted by the journal verified the majority of these concerns and found that some results were not supported by the available data.

The authors responded to an inquiry by the publisher by addressing the reported concerns and providing an amended manuscript which included the missing study design and methodological details. Crucially, the corrected manuscript included changes which fundamentally altered the results and conclusions of the article. All parties have agreed that the article must be retracted because the results and conclusions presented in the article are no longer considered reliable. The authors The authors were informed of the retraction.

